# Phenolic Compounds from Cherries and Berries for Chronic Disease Management and Cardiovascular Risk Reduction

**DOI:** 10.3390/nu16111597

**Published:** 2024-05-23

**Authors:** Filomena Carvalho, Radhia Aitfella Lahlou, Luís R. Silva

**Affiliations:** 1 SPRINT—Sport Physical Activity and Health Research & Innovation Center, Instituto Politécnico da Guarda, 6300-559 Guarda, Portugal; filomenacarvalho@ipg.pt (F.C.); radhialahlou@ipg.pt (R.A.L.); 2 CICS-UBI—Health Sciences Research Center, University of Beira Interior, 6201-506 Covilhã, Portugal; 3 CERES, Department of Chemical Engineering, University of Coimbra, 3030-790 Coimbra, Portugal

**Keywords:** cherries, berries, red fruits, phenolic compounds, cardiovascular diseases

## Abstract

Cardiovascular diseases (CVDs) are a leading cause of mortality worldwide. Therefore, there is increasing interest in dietary interventions to reduce risk factors associated with these conditions. Cherries and berries are rich sources of bioactive compounds and have attracted attention for their potential cardiovascular benefits. This review summarises the current research on the effects of cherry and berry consumption on cardiovascular health, including in vivo studies and clinical trials. These red fruits are rich in phenolic compounds, such as anthocyanins and flavonoids, which have multiple bioactive properties. These properties include antioxidant, anti-inflammatory, and vasodilatory effects. Studies suggest that regular consumption of these fruits may reduce inflammation and oxidative stress, leading to lower blood pressure, improved lipid profiles, and enhanced endothelial function. However, interpreting findings and establishing optimal dosages is a challenge due to the variability in fruit composition, processing methods, and study design. Despite these limitations, the evidence highlights the potential of cherries and berries as components of preventive strategies against CVD. Further research is needed to maximise their health benefits and improve clinical practice.

## 1. Introduction

Cardiovascular diseases (CVDs) are responsible for an estimated 18.6 million deaths worldwide, each year [1]. The World Health Organization (WHO) defines CVD as a group of conditions that affect the heart and blood vessels, including cerebrovascular disease, congenital heart disease, rheumatic heart disease, peripheral artery disease, deep vein thrombosis, and pulmonary embolism [2]. While there are many pharmaceutical treatments for cardiovascular problems, long-term use of current medications can result in several adverse effects, including haemorrhagic stroke, rhabdomyolysis, and renal failure [3].

Diets that are rich in naturally occurring bioactive compounds have attracted considerable interest due to their ability to maintain or improve cardiovascular health [2,4]. Due to their high content of bioactive compounds, high consumption of fruits and vegetables has been associated with a reduced incidence of CVD [5,6]. Red fruits, such as cherries and berries are a rich source of nutrients and are low in calories (Table 1). They are rich in vitamins, minerals, and antioxidants, and are low in fat, containing mostly unsaturated fat, making them a good choice for promoting heart health. Additionally, they are also a good source of fibre. Including a variety of these fruits in a diet provides a wide range of nutrients to promote overall health and well-being [7].

Red fruits contain a significant number of phenolic compounds, such as phenolic acids (hydroxycinnamic and hydroxybenzoic acids), flavonoids (flavanols, flavonols, and anthocyanins), and stilbenes (resveratrol) [7]. These secondary metabolites have been extensively studied and are characterised by their strong antioxidant capacity and a spectrum of bioactive properties, including anti-proliferative, anti-diabetic, anti-cancer, antimicrobial, anti-inflammatory, and antiviral effects [9].

Therefore, this review aims to provide a comprehensive synthesis of the current research evidence on cherry and berry consumption, as observed by in vivo studies and clinical trials, with a focus on their potential impact on CVDs. The aim is to shed light on the complex relationships between the consumption of red fruit and cardiovascular health, exploring both the established benefits and potential areas for further investigation. The articles were searched using the Web of Science database, to ensure a comprehensive coverage of the literature. We limited our search to studies performed from 2010 until now, aiming to focus on the most recent research. To review the chemical composition of the fruits, the keywords used were “phenolic” and “HPLC” and the species of the fruits in order to analyse the qualitative and quantitative presence of the phenolic compounds. For research related to the impact of fruit consumption on cardiovascular health, the keywords used were the name of the fruits followed by “cardiovascular”. The search was further refined by checking the species of the fruit to ensure the relevance and specificity of the articles.

## 2. Cardiovascular Disease

**Figure 1:**
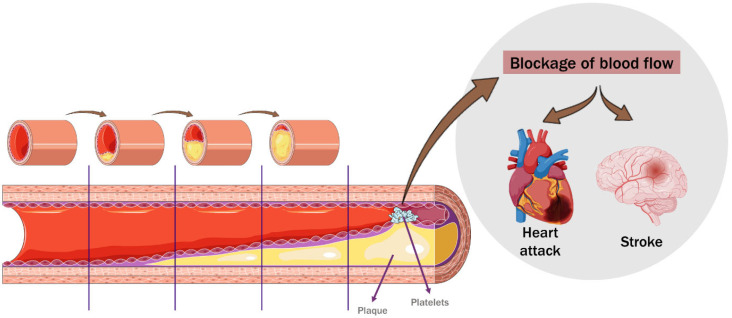
Atherosclerosis progression.

CVD is the leading cause of death worldwide, with an estimated number of 18.6 million deaths in 2019 [1]. In total, 85% of these deaths were caused by heart attacks and strokes, which are typically sudden occurrences mostly caused by a blockage that stops blood from reaching the brain or heart. This blockage is often caused by an accumulation of fatty deposits on the inner walls of the arteries that supply blood to the brain or heart [10]. Atherosclerosis is the primary cause of CVD, being characterised by the accumulation of lipids and inflammation in the large arteries (see Figure 1), and it is a multifactorial disorder caused and exacerbated by risk factors, such as dyslipidaemia, oxidative and inflammatory stress, diabetes mellitus, hypertension, smoking, aging, and genetic abnormalities [11,12]. In 2020, diseases of the circulatory system caused approximately 1.70 million deaths in the European Union (EU) [13].

CVDs cover a range of conditions, including coronary heart disease (affects the blood vessels that supply the heart muscle), cerebrovascular disease (affects the blood vessels that supply the brain), peripheral arterial disease (affects the blood vessels that supply the arms and legs), and other CVDs, such as rheumatic heart disease (caused by streptococcal bacteria that damages the heart muscle and heart valves), congenital heart disease (heart structural malformations present from birth), cardiomyopathies, and cardiac arrhythmias [10,14]. Approximately 80% of CVD cases are caused by behavioural risk factors like smoking, poor diet, and physical inactivity. Intermediate risk factors for CVD, such as obesity, high blood pressure, blood sugar, and cholesterol, can be directly caused by behavioural risk factors [10].

The economic impact of CVD continues to grow, making it the most expensive condition, surpassing both diabetes and Alzheimer’s disease [15]. It is estimated that CVD had a $906 billion global economic burden in 2015, which is projected to increase by 22% by 2030 [16]. The epidemiological transition and increased incidence of lifestyle-related disorders, such as type 2 diabetes and hypertension, have contributed to the growing burden of CVD worldwide [17]. CVD is the leading cause of death and morbidity in people with diabetes [18]. The incidence of CVD in young adults is increasing and has been linked to several variables, including obesity, overweight, tobacco use, and unhealthy diet, suggesting that the disease is not confined to a specific age group [19]. The impact of CVD is also observed in different geographical regions, as studies have reported different prevalence rates of CVD in different populations, including rural and urban settings [20]. Studies have shown that the prevalence of CVD varies across European countries, with some regions experiencing higher rates of CVD than others. For instance, Turkey has been reported to have the highest rate of premature CVD in Europe [21]. In addition, the prevalence of CVD has been reported to be higher in women than in men in Europe [22].

Common symptoms of CVD include chest pain, shortness of breath, and fatigue [23]. However, it is important to note that the prodromal symptoms of CVD may be significantly more atypical in women than in men, suggesting the need for increased vigilance in the cardiovascular evaluation of women with non-anginal symptoms [24]. Depressive symptoms have been associated with adverse cardiovascular events, particularly in patients with coronary heart disease, with the association being largely explained by behavioural factors, particularly physical inactivity [25].

The treatment of CVD involves a variety of approaches to manage the condition. Novel treatments include smoking cessation therapy, antiplatelet, antithrombotic, lipid-lowering, antihypertensive agents, nonpharmacological therapy for nonvalvular atrial fibrillation, and more traditional treatments, such as caloric restriction [26]. However, the most important factor for CVD management is prevention through healthy lifestyle practices. According to the WHO, reducing salt intake, increasing fruit and vegetable intake, smoking cessation, regular physical activity, and alcohol abstinence have been shown to reduce the risk of CVD [10].

## 3. Phenolic Composition of Cherries and Berries

Phenolic compounds are a class of bioactive molecules that have been extensively studied and have numerous health benefits. These compounds, formed by multiple hydroxyl groups on aromatic rings, are secondary metabolites of plants, involved in the protection against ultraviolet radiation or aggression by pathogens [27]. They are used in the pharmaceutical industry for the treatment of various diseases, in the food industry as additives, natural preservatives, and colourings, and in the cosmetic industry for their antimicrobial, antioxidant, and anti-inflammatory properties, as well as protection against UV radiation [28].

Certain fruits, including cherries, berries, grapes, apples, and pears can contain up to 200–300 mg of polyphenols per 100 g of fresh weight. Additionally, polyphenols can be found in beverages, such as tea, wine, and coffee with approximately 100 mg per serving. Other foods such as chocolate, cereals, and dried legumes may also contain these compounds [29]. The daily intake of polyphenols obtained from a normal diet rich in fruits, vegetables, and plant-based beverages is approximately 1 g, with variations depending on the number of polyphenol-rich beverages consumed [30]. Numerous epidemiological studies have investigated the correlation between the consumption of polyphenol-rich foods and chronic diseases, suggesting that these chemicals have a preventive effect against CVDs [31].

The generality of plants contains phenolics, with the most prevalent classes being phenolic acids, flavonoids, and tannins [30]. Phenolic acids can be further divided into two groups: hydroxycinnamic acids (C6–C3) and hydroxybenzoic acids (C6–C1). Most edible plants include very little hydroxybenzoic acid (gallic, salicylic, and vanillic acids), with the exception of some red fruits, onions, and black radishes. Hydroxycinnamic acids (*p*-coumaric, caffeic, ferulic, and sinapic acids) are the most present phenolics in highly consumed foods, like coffee, cereals, and fruits [31].

The second major class of polyphenols found in the human diet is called flavonoids [31]. Flavonoids can be classified into anthocyanidins (found in berries and wine), flavones (herbs), isoflavones (soyabean), flavonols (tea, broccoli, onions, and tomatoes), flavanones (citrus fruits and juices), and flavan-3-ols (cocoa, tea, wine, and fruits) [27].

Anthocyanins are pigments that appear as red in acidic conditions and as blue in alkaline conditions, and exist in flowers, fruits, and tubers of plants. Cyanidin, malvidin, delphinidin, peonidin, petunidin, and pelargonidin are the most common types; these coloured pigments are powerful antioxidants, and they can be found in berries, blackcurrants, and other red to blue coloured fruits [32]. The stability of anthocyanins is affected by pH, light, temperature, and their structure [33].

Tannins are high-molecular-weight compounds with diverse chemical structures, which can be divided into condensed tannins or proanthocyanidins, complex tannins, and hydrolysable tannins (gallic acid or ellagic acid). Foods in which they can be found include fruits, vegetables, legume seeds, cereals, and beverages such as wine, tea, cocoa, nuts and cider [34].

Table 2 provides an overview of the main compounds identified in the studied fruits. Both tart cherry and sweet cherry exhibit similarity in their hydroxycinnamic acid profiles, sharing compounds such as 3,5-dicaffeoylquinic acid, 3-*p*-coumaroylquinic acid, and chlorogenic acid, indicating a close botanical relationship between the two species. Blackberries have a diverse collection of hydroxycinnamic acids, indicating their distinctive phytochemical fingerprint. Blueberries contain several caffeic acid derivatives, contributing to their antioxidant potential [35]. Among the hydroxybenzoic acids, gallic acid stands out and is found in sweet cherry, blackberry, blueberry, and red raspberry. This compound may contribute to the unique health properties associated with these fruits, such as protection against gastrointestinal, metabolic, neuropsychological, and CVD [36,37,38,39].

The anthocyanin content presents a rich variety of compounds unique to each fruit. Tart cherry contains cyanidin-3-*O*-glucoside and related derivatives, highlighting its potential health-promoting effects, as this compound has been intensively studied as one of the most abundant anthocyanins; it is widely distributed in red and blue fruits [40] and shows health benefits in humans with diseases such as CVD and cancer [41,42]. Sweet cherry shares common anthocyanins with tart cherry but exhibits quercetin-3-4′-di-O-glycoside. Blackberries show a spectrum of cyanidin and malvidin derivatives. Blueberries are characterised by numerous anthocyanin derivatives, which contribute to their attractive colour spectrum, which is one of the most important sensory characteristics of berry products [43]. Strawberries and red raspberries show variations in anthocyanin composition, with unique glycosides and derivatives.

**Figure 2:**
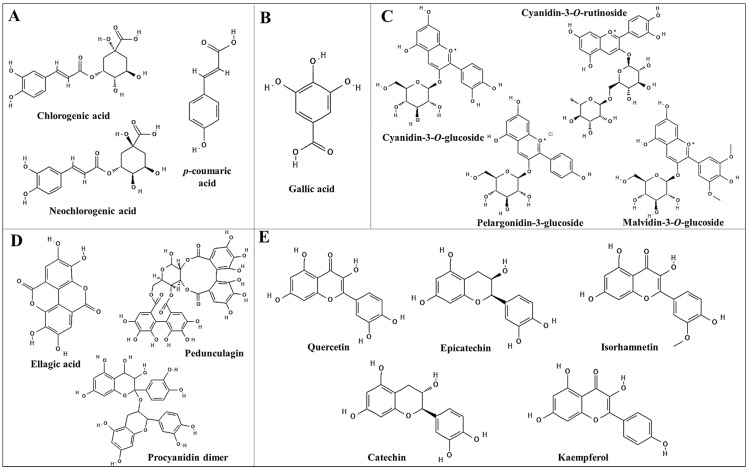
Chemical structures of the most common compounds found in cherries and berries. (**A**) Hydroxycinnamic acids; (**B**) Hydroxybenzoic acids; (**C**) Anthocyanins; (**D**) Tannins; (**E**) Flavonoids other than anthocyanins.

In the flavonoid category, tart cherry and sweet cherry share common flavonoids, including catechin, epicatechin, isorhamnetin, and quercetin derivatives. Blackberries have a wide range of flavonoids, while blueberries and strawberries have a profile that includes catechin, epicatechin, kaempferol, and quercetin derivatives. Blackberries, blueberries, strawberries, and red raspberries have different tannin compositions. Blackberries are rich in casuarinin, ellagic acid, and procyanidin dimer. Blueberries show ellagic acid and procyanidin dimer. A strawberry’s tannin profile includes agrimoniin, ellagic acid derivatives, glucogallin, and various procyanidins. Red raspberries display ellagic acid derivatives, procyanidin dimer, and sanguiin derivatives. Figure 2 displays the chemical structure of the most common compounds found in the fruits studied.

The diversity observed across the fruits highlights the importance of consuming a variety of fruits to maximise the intake of different bioactive compounds associated with well-being. The plant content and composition of polyphenolic compounds are variable, as they can be influenced by factors, such as growing conditions and climate, plant variety, processing, and analytical methods used for identification [44]. Quantitatively speaking, these factors contribute to a wide range of values reported in different studies, which also use different units of measurement, leading to difficulty in assessing and comparing the levels of polyphenols.

Considering the diversity and health-promoting potential of the compounds present in the cherries and berries analysed, the next chapter discusses the research that has been carried out on the administration or consumption of different preparations of these fruits and the relationship between their composition and their CVD-preventing mechanisms.

**Table 2:** Phenolic compounds found in tart cherry, sweet cherry, blackberry, blueberry, strawberry, and red raspberry fruits, by class.

Compound Class	Fruit (Species)	Reported Compounds	References
Hydroxycinnamic acids and derivates	Tart cherry (*Prunus cerasus*)	3,5-dicaffeoylquinic acid,	[45,46]
		3-*p*-coumaroylquinic acid,	
		4-*p*-coumaroylquinic acid,	
		chlorogenic acid,	
		neochlorogenic acid	
	Sweet cherry (*Prunus avium*)	3,5-dicaffeoylquinic acid,	[45,46,47]
		3-*p*-coumaroylquinic acid,	
		4-*p*-coumaroylquinic acid,	
		chlorogenic acid,	
		neochlorogenic acid,	
		*p*-coumaric acid	
	Blackberry (*Rubus* spp.)	3-*O*-caffeoylquinic acid,	[48,49,50]
		3-*O*-caffeoylquinic acid dimer,	
		caffeic acid,	
		ferulic acid,	
		ferulic acid-*O*-hexoside,	
		neochlorogenic acid,	
		*p*-coumaric acid,	
		sinapic acid,	
		*trans*-cinnamic acid	
	Blueberry (*Vaccinium corymbosum*)	Caffeic acid,	[51,52]
		caffeoylhexose,	
		chlorogenic acid,	
		ferulic acid,	
		feruloylhexose,	
		malonyl-caffeoylquinic acid,	
		malonyl-dicaffeoylquinic acid	
	Strawberry (*Fragaria* × *ananassa*)	Cinnamoyl-glucose,	[53,54,55]
		cinnamoyl xylosylglucose,	
		O-*p*-coumaroylhexose,	
		1-*O*-feruloylglucose,	
		1-*O*-trans-cinnamoyl-*β*-glucose,	
		*p*-coumaric acid,	
		*p*-coumaric acid derivatives,	
		*p*-coumaroyl hexose	
	Red raspberry (*Rubus idaeus*)	Caffeic acid,	[56,57,58]
		caffeic acid-*O*-glucoside,	
		chlorogenic acid	
Hydroxybenzoic acids and derivates	Tart cherry (*Prunus cerasus*)	-	
	Sweet cherry (*Prunus avium*)	Gallic acid	[47]
	Blackberry (*Rubus* spp.)	Gallic acid,	[48,49,50]
		vanillic acid,	
		protocatechuic acid	
	Blueberry (*Vaccinium corymbosum*)	Gallic acid	[51]
	Strawberry (*Fragaria* × *ananassa*)	-	
	Red raspberry (*Rubus idaeus*)	Gallic acid,	[57,58]
		lambertianin C	
Anthocyanins and derivatives	Tart cherry (*Prunus cerasus*)	Cyanidin-3-*O*-glucoside,	[45,46,59]
		cyanidin-3-*O*-xylosyl-rutinoside,	
		cyanidin-3-*O*-rutinoside,	
		cyanidin-3-sophoroside,	
		cyanidin-3-*O*-glucosy-lrutinoside,	
		delphinidin-3-*O*-rutinoside,	
		peonidin-3-*O*-rutinoside	
	Sweet cherry (*Prunus avium*)	Cyanidin-3-*O*-glycoside,	[45,46,47]
		cyanidin-3-*O*-glucoside,	
		cyanidin-3-*O*-glucosyl-rutinoside,	
		cyanidin-3-*O*-rutinoside,	
		peonidin-3-*O*-rutinoside,	
		quercetin-3-4′-di-*O*-glycoside	
	Blackberry (*Rubus* spp.)	Cyanidin-3-*O*-hexoside,	[48]
		cyanidin-3-*O*-pentoside,	
		cyanidin-3-*O*-acetylglucoside,	
		cyanidin-3-*O*-arabinoside,	
		cyanidin-3-*O*-glucoside,	
		cyanidin-3-*O*-xyloside,	
		delphidin-3-*O*-glucoside,	
		malvidin-3-*O*-glucoside	
	Blueberry (*Vaccinium corymbosum*)	Cyanidin-3-*O*-arabinoside,	[51,52]
		cyanidin-3-*O*-galactoside,	
		cyanidin-3-*O*-glucoside,	
		delphinidin-3-*O*-arabinoside,	
		delphinidin-3-*O*-galactoside,	
		delphinidin-3-*O*-glucoside,	
		malvidin-3-*O*-arabinoside,	
		malvidin-3-*O*-galactoside,	
		malvidin-3-*O*-glucoside,	
		peonidin-3-*O*-arabinoside,	
		peonidin-3-*O*-galactoside,	
		peonidin-3-*O*-glucoside,	
		petunidin-3-*O*-arabinoside,	
		petunidin-3-*O*-galactoside,	
		petunidin-3-*O*-glucoside	
	Strawberry (*Fragaria* × *ananassa*)	Cyanidin-3-*O*-glucoside,	[53,54,55]
		cyanidin-3-*O*-glucosyl-rutinoside,	
		cyanidin-3-*O*-hexoside,	
		cyanidin-3-*O*-pentoside,	
		cyanidin-3-malonylglucoside,	
		pelargonidin-3,5-diglucoside,	
		pelargonidin-3-acetylglucoside,	
		pelargonidin-3-galactoside,	
		pelargonidin-3-glucoside,	
		pelargonidin-3-rutinoside,	
		pelargonidin hexosides	
	Red raspberry (*Rubus idaeus*)	Cyanidin-3-*O*-glucoside,	[56,58]
		cyanidin-3-*O*-glucosyl-rutinoside,	
		cyanidin-3-*O*-rutinoside,	
		cyanidin-3-*O*-sambubioside,	
		cyanidin-3-*O*-sophoroside,	
		pelargonidin-3-*O*-glucoside,	
		pelargonidin-3-*O*-sophoroside	
Flavonoids other than anthocyanins	Tart cherry (*Prunus cerasus*)	Catechin,	[45,46]
		epicatechin,	
		isorhamnetin,	
		kaempferol-3-*O*-hexoside,	
		kaempferol-3-*O*-rutinoside,	
		quercetin,	
		quercetin-3-*O*-glucoside,	
		quercetin-3-*O*-glucosyl-rutinoside,	
		quercetin-3-*O*-rhamnoside,	
		quercetin-3-*O*-rutinoside	
	Sweet cherry (*Prunus avium*)	Catechin,	[45,46,47]
		epicatechin,	
		isorhamnetin,	
		isorhamnetin-3-*O*-hexoside,	
		kaempferol-3-*O*-hexoside,	
		kaempferol-3-*O*-rutinoside,	
		quercetin,	
		quercetin-3-*O*-glucoside,	
		quercetin-3-*O*-glucosyl-rutinoside,	
		quercetin-3-*O*-rhamnoside,	
		quercetin-3-*O*-rutinoside	
	Blackberry (*Rubus* spp.)	3-Hydroxy-3-MG-quercetin-*O*-hexoside,	[48,49,50]
		catechin,	
		epicatechin,	
		isorhamnetin,	
		kaempferol-3-*O*-coumaroylglucoside,	
		kaempferol-3-*O*-galactoside,	
		kaempferol-3-*O*-hexoside,	
		kaempferol-*O*-acetylhexoside,	
		quercetin,	
		quercetin-3-*O*-galactoside,	
		quercetin-3-*O*-glucoside,	
		quercetin-3-*O*-glucuronide,	
		quercetin-*O*-acetylhexoside,	
		quercetin-3-*O*-glucuronide,	
		quercetin-*O*-hexoside,	
		quercetin-*O*-pentoside,	
		quercetin-3-*O*-rutinoside	
	Blueberry (*Vaccinium corymbosum*)	Catechin,	[51,52]
		epicatechin,	
		quercetin,	
		quercetin 3-*O*-rutinoside	
	Strawberry (*Fragaria* × *ananassa*)	Catechin,	[53,54,55]
		dihydroflavanone-*O*-coumaroylhexoside,	
		dihydrokaempferol,	
		isorhamnetin,	
		isorhamnetin-*O*-acetylhexoside,	
		isorhamnetin-*O*-deoxyhexoside,	
		kaempferol,	
		kaempferol-3-coumaroylglucoside,	
		kaempferol-3-glucoside,	
		kaempferol-3-glucuronide,	
		kaempferol-3-hexoside,	
		kaempferol-*O*-acetylhexoside,	
		quercetin-3-*O*-glucoside,	
		quercetin-3-*O*-glucoside derivative,	
		quercetin-3-glucuronide,	
		quercetin-3-malonylglucoside,	
		quercetin-*O*-pentoside,	
		taxifolin-3-*O*-*β*-arabinoside	
	Red raspberry (*Rubus idaeus*)	Brevifolincarboxylic acid,	[56,57,58]
		catechin,	
		catechin derivative,	
		epicatechin,	
		epigallocatechin,	
		kaempferol,	
		kaempferol-3-glucoside,	
		kaempferol-3-glucuronide,	
		quercetin,	
		quercetin-3-glucoside,	
		quercetin-3-glucuronide,	
		quercetin-3-*O*-galactoside,	
		quercetin-3-*O*-glucoside,	
		quercetin-3-*O*-rhamnosyl-galactoside,	
		quercetin-3-*O*-galactoside,	
		quercetin-3-*O*-rutinoside,	
		tiliroside	
Tannins and derivatives	Tart cherry (*Prunus cerasus*)	-	
	Sweet cherry (*Prunus avium*)	-	
	Blackberry (*Rubus* spp.)	Casuarinin,	[48,49,50]
		ellagic acid,	
		ellagic acid-*O*-glucuronide,	
		ellagic acid-*O*-hexoside,	
		ellagic acid-*O*-pentoside,	
		pedunculagin I,	
		procyanidin dimer	
	Blueberry (*Vaccinium corymbosum*)	Ellagic acid,	[51,52]
		procyanidin dimer	
	Strawberry (*Fragaria* × *ananassa*)	Agrimoniin,	[53,54,55]
		davuriicin D2,	
		davuriicin M1,	
		digalloyl-tetraHHDP-diglucose,	
		ellagic acid,	
		ellagic acid deoxyhexoside,	
		ellagic acid pentoside,	
		galloyl-diHHDP-glucose,	
		glucogallin,	
		methyl ellagic acid deoxyhexoside,	
		pedunculagin,	
		potentillin,	
		procyanidin dimer,	
		procyanidin pentamer,	
		procyanidin trimer,	
		tetragalloylglucose	
	Red raspberry (*Rubus idaeus*)	Ellagic acid,	[56,57,58]
		ellagic acid pentoside,	
		ellagic acid-4-*O*-acetylxyloside,	
		galloyl-diHHDP-glucose,	
		procyanidin dimer,	
		sanguiin H-2,	
		sanguiin H-6,	
		sanguiin H-6 isomer,	
		sanguiin H-10 isomer	

-: not reported.

## 4. The Role of Cherry and Berry Consumption in Cardiovascular Disease

Several in vivo studies and clinical trials have demonstrated the effects of cherry and berry consumption on various health parameters associated with CVD or its risk factors (Table 3 and Table 4). These effects include decreases in blood pressure, improvement in lipid profiles, reduction in inflammation and oxidative stress, and improvement in endothelial function, among others.

The risk of CVD is increased by hypertension, particularly isolated systolic hypertension [60]. Mean reductions in brachial systolic blood pressure of at least 5.6 mmHg over five years have been associated with a 25–40% and 20–25% reduced risk of coronary heart disease and stroke, respectively [61]. An in vivo study analysed the effects of tart cherry consumption in obese rats. The study demonstrated that juice and seed supplementation caused a reduction in systolic blood pressure. The authors attribute this effect to the vasodilatory capacity of anthocyanins [62]. Cardiovascular research has examined rats as genetic models of obesity, including the Zucker obese rat, the ob/ob rat, and the spontaneously hypertensive obese rat [63,64,65].

Several clinical trials have demonstrated that the consumption of these fruits can reduce blood pressure. Some studies refer to the blood pressure-lowering effects of concentrated tart cherry juices. The clinical trials by Desai et al. 2019 and 2021 with metabolic syndrome adults demonstrated a reduction in blood pressure of 11 mmHg and 5 mmHg, respectively, 2 h after the consumption of concentrated cherry juice, and after 6 days of consumption [66,67]. The metabolic syndrome has been identified as a predictor of long-term total and cardiovascular mortality, emphasizing its clinical relevance in CVD risk assessment [61]. In men with early hypertension, the consumption of tart cherry concentrated juice had a blood pressure-lowering effect similar to that obtained with antihypertensive drugs in mildly hypertensive subjects; this effect was associated with peak plasma protocatechuic acid and vanillic acid metabolites [68]. According to the research on the chemical composition of tart cherry, the authors did not find a record of the presence of these compounds in fruits (Table 2). However, Obón et al. found that vanillic acid is present in tart cherry juice [69]. In fact, in vitro studies have demonstrated that hydroxybenzoic acids influence the behaviour of vascular smooth muscle cells [70]. As for sweet cherries, only one clinical trial has proven that a sweet cherry drink was able to significantly reduce the systolic blood pressure of obese adults, demonstrating the fruit’s potential to reduce hypertension in subjects with a body mass index (BMI) ≥ 35 [71]. Freeze-dried blueberries have been shown to reduce systolic blood pressure in sedentary adults and in obese adults with metabolic syndrome when consumed daily [72,73], and freeze-dried strawberries reduced the blood pressure in adults with hypercholesterolemia [74]. 

Endothelial function is a key marker of cardiovascular health. Damage to the endothelium can disrupt the balance of blood flow regulation, leading to endothelial dysfunction, the underlying condition for atherosclerosis, hypertension, and other CVDs [75,76]. Oxidative stress plays a role in the pathophysiology of atherosclerosis by disrupting the coupling of endothelial nitric oxide (NO) synthase, causing endothelial dysfunction. It also damages endothelial proteins, lipids, and DNA [77]. An in vivo study conducted on hypertensive rats demonstrated a reduction in systolic blood pressure after one week of administering an ethyl acetate extract of red raspberry, revealing a gradual reduction over the week [78]. At the same time, the extract reduced serum endothelin and increased serum nitric oxide, leading the authors to suggest that the systolic blood pressure-lowering effects of red raspberry are associated with the maintenance of the NO/ET (nitric oxide/endothelins) balance. Oxidative stress appears to affect vascular function through the regulation of endothelin-1 (ET-1) and the depletion of bioavailable NO [79]. Hypertensive patients have been found to have low NO levels, demonstrating the importance of NO in blood pressure control [80]. The phenolic compounds in the fruits seem to be responsible for these effects. Lazzè et. al described that the anthocyanidins (aglycon forms of anthocyanins) delphidin and cyanidin decrease the production of ET-1 in human umbilical vein endothelial cells, showing an inhibitory effect on the protein and mRNA levels of ET-1. On the other hand, the compounds increased the protein levels of endothelial nitric oxide synthase (eNOS) [79]. Cyanidin-3-glucoside, an anthocyanin, has been found to increase nitric oxide synthesis by upregulating the expression of endothelial nitric oxide synthase (eNOS) [81]. The production of nitric oxide by endothelial nitric oxide synthase influences blood pressure and is therefore crucial for cardiovascular homeostasis [81]. 

Currently, hypertension can be treated with a variety of synthetic angiotensin-converting enzyme (ACE) inhibitors, another enzyme that affects vascular function, either by converting angiotensin I to angiotensin II, which causes vasoconstriction [82], or by degrading bradykinin, a potent vasodilator [83]. However, these drugs may have negative side effects [84]. Bioactive compounds present in these fruits, such as ferulic, *p*-coumaric, *trans*-cinnamic, caffeic, gallic, ellagic, vanillic and protocatechuic acids, quercetin, kaempferol, and epicatechin, have been shown to inhibit ACE’s activity [85], making them potential natural ACE inhibitors, while reducing the negative effects of synthetic options. 

Several other studies have shown that cherries and berries can improve endothelial function. Meister et al. (2023) evaluated changes in the protein expression of pro-oxidant and inflammatory markers as an indicator of microvasculature in rats exposed to e-cigarettes. They concluded that blackberry consumption attenuated vascular oxidative stress caused by e-cigarette exposure [86]. Woolf et al. (2023) found improvements in endothelial function in postmenopausal women with hypertension after 12 weeks of blueberry consumption; they attributed this effect to a reduction in oxidative stress and linked it to polyphenol metabolites found in plasma [87]. 

Some studies have proposed different mechanisms that may be behind these antioxidant and anti-inflammatory effects. Martinelli et al. (2022) found that the antioxidant properties of tart cherry were associated with a reduction in protein carbonyls levels (markers of protein oxidation) and, in particular, 4-HNE (4-hydroxynonenal), which is associated with myocardial damage; they associated the anti-inflammatory effect of the fruit to a reduction in retroperitoneal IL-6 and TNF-α mRNA expression, NF-κB activity, and plasma IL-6 and TNF-*α* levels [62].

The consumption of blueberry powder also reduces the mRNA levels of inflammatory markers TNF-*α*, IL-6, and TLR4 in the monocytes of adults with metabolic syndrome [88]. In obese subjects, intake of dark sweet cherries for 30 days reduced IFN*y* levels by 30% and showed a trend towards a 5% reduction in MCP-1. These are two pro-inflammatory cytokines involved in the obesity-induced inflammatory response, associated with increased CVD, and their suppression may be considered a dietary approach to reduce obesity complications [71]. CRP, another marker of inflammation, was reduced by sweet cherry supplementation in obese rats. In the same study, the activity of the antioxidant enzymes GPx, GR, and CAT was increased by the consumption of the fruit, indicating a reduction in oxidative stress [89].

Rangel-Huerta et al. conducted a review and found that dietary phenolic compounds, including catechols, stilbenes, anthocyanins, catechins, flavanols and flavonols, isoflavones, and procyanidins have shown a wide range of anti-inflammatory and antioxidant effects through the control of various mentioned biomarkers, such as CRP, TNF-*α*, and IL-6, among many others [90].

Frequent consumption of a high-fat diet can increase the risk of obesity, which is strongly associated with cardiovascular disorders [91]. Dysregulated lipid metabolism leads to excessive accumulation of lipids in the liver and adipose tissue, which leads to the synthesis of pro-inflammatory cytokines, and contributes to chronic inflammation, while a high-fat diet decreases antioxidant enzyme activity and induces oxidative stress [92,93,94]. Elevations in indicators such as triglycerides, cholesterol, and low-density lipoprotein cholesterol (LDL-C) are of concern, as they are risk factors for the development of CVD [95]. Some of these phenolic-rich fruits appear to have an effect on the lipid profile in vivo and in humans (Table 3 and Table 4).

A study has reported that the daily consumption of 500 g of strawberries for one month reduced the total cholesterol (8.8%), LDL-C (13.7%), and triglyceride levels (20.8%) of healthy adults [95]. In addition, the consumption of blueberry and raspberry cookies caused a significant reduction in LDL-C in healthy women, which the author attributed to a synergistic effect between the intake of dietary fibre, unsaturated fatty acids, and a high dose of anthocyanins [96]. Previously, anthocyanins, including 3-*O*-*β*-glucosides, 3-*O*-*β*-galactosides, and 3-*O*-*β*-arabinosides of cyanidin, delphinidin, petunidin, peonidin, malvidin, and delphinidin have demonstrated an ability to increase HDL-C and decrease LDL-C in dyslipidaemic subjects after 12 weeks of supplementation [97]. Anthocyanins such as malvidin and its derivatives can reduce the expression of HMG-CoA reductase, an enzyme that catalyses an important step in the synthesis of cholesterol [98]. Anthocyanins, namely cyanidin 3-*O*-*β*-glucosides, have also been shown to inhibit the cholesteryl ester transfer protein (CETP) [97], a protein involved in the transfer of cholesteryl esters from HDL to other plasma lipoprotein fractions, and thus its suppression may increase cholesterol levels in the potentially protective HDL fraction while lowering them in the proatherogenic non-HDL fractions [99]. Research has demonstrated that anthocyanins and hydroxycinnamic acids, mainly caffeic acid, ferulic acid, and chlorogenic acid, reduce the activity of lipolytic enzymes, such as pancreatic lipase, and may inhibit fat absorption in the intestinal lumen [100,101]. 

According to Burton-Freeman et al. (2010), the consumption of a freeze-dried strawberry beverage was able to decrease LDL-C oxidation after 10 days in overweight and hyperlipidaemic adults [102]. The oxidative conversion of LDL-C into oxidized LDL (ox-LDL) is one of the main events in the development of atherosclerosis, and dietary polyphenols have been shown to inhibit this process [103]. 

Dziadek et al. (2019) found that the consumption of sweet cherries improved the lipid profile of rats on a high-fat cholesterol diet via an effect on lipid metabolism derived from the regulation of the expression of certain genes: decreased expression of Fasn, Acaca, Scd1, Mlxipl, and Srebf1 in liver and adipose tissue and increased expression of Cpt1a and Ppar-*α* in the liver [89]. Acaca and Fasn are involved in lipogenesis, regulating the conversion of acetyl-CoA to malonyl-CoA and malonyl-CoA to palmitate, respectively [104]; Scd1 and Srebf1 regulate these enzymes and induce Scd1, which controls fatty acids synthesis [105]. PPAR-*α* is responsible for the regulation of genes involved in fatty acid oxidation, including Cpt1a, which regulates an enzyme with a crucial role in mitochondrial *β*-oxidation [106].

As we have seen, cherries and berries offer significant benefits in reducing cardiovascular risk by targeting obesity. Moreover, they have been reported to also target type 2 diabetes, which is commonly associated with obesity. Perez-Verdaguer et al. (2016) have reported that flavonoids, compounds in which these fruits are rich, are known for their ability to block ion channels, including the voltage-gated potassium channels Kv1.3. The inhibition of Kv1.3 channels plays a crucial role in metabolic regulation. Blocking or downregulating these channels can enhance peripheral insulin sensitivity. This increased sensitivity improves sugar metabolism, contributing to weight loss and improved locomotor activity and mass-specific metabolism [107]. Together, these effects may help reduce the risk factors associated with cardiovascular diseases.

**Table 3:** In vivo studies with the administration of cherries and berries and their effects on cardiovascular disease-related factors.

Fruit (Species)	Animal Model	Fruit Preparation	Procedure	Main Results	Ref.
Tart cherry (*Prunus cerasus*)	Dietary-induced obese rats	Seed powder or seed powder + juice	Supplementation with seed powder (1 mg/g of fat) or seed powder (1 mg/g of fat) + juice (1 mg AC), daily for 17 weeks	Reduction in systolic blood pressure, oxidative stress, and inflammation	[62]
	Dietary-induced obese rats	Seed powder or seed powder + juice	Supplementation with seed powder (1 mg/g of fat) or seed powder (1 mg/g of fat) + juice (1 mg AC), daily for 17 weeks	No effects in accumulation of visceral fat Reduction in inflammatory markers	[108]
Sweet cherry (*Prunus avium*)	Dietary-induced obese rats	Freeze-dried fruit	Supplementation of 5 or 10% of freeze-dried cherries, daily for 12 weeks	Reduction in body weight, oxidative stress, and inflammation Improvement in liver function and lipid profile	[89,109]
Blackberry (*Rubus* spp.)	Dietary-induced obese rats	Freeze-dried fruit	High-fat and sucrose diet supplemented with 10% blackberry or 10% blackberry + raspberry	Reduction in inflammation and oxidative stress (alteration of redox proteins from the myocardium), when combined with raspberry	[110]
	Atherosclerosis rat models	Freeze-dried powder	High-fat diet supplemented with 2% of powder, ad libitum for 5 weeks	Reduction in plaque accumulation, senescence-associated-β-galactosidase, and Nox1 expression in the aorta of male rats No effects on lipid profile	[111]
	Ovariectomized rats	Freeze-dried powder	Diet supplemented with 5 or 10% of powder, daily for 100 days	Reduction in ovariectomy-induced weight gain and downregulation of inflammation-related genes (with high dose) Improvement in lipid profile	[112]
	Rats exposed to e-cigarette vapor	Freeze-dried powder	Diet supplemented with 5% of powder, daily for 16 weeks	Mitigated the increase of oxidative stress markers Reduced endothelial dysfunction No effects on serum antioxidant capacity	[86]
	Dietary-induced obese rats	Freeze-dried powder	High-fat and sucrose diet supplemented with 10% blackberry or 10% blackberry + raspberry, daily for 20 weeks	Reduction in aortic oxidative stress and oxidative burden to the endothelium	[113]
Blueberry (*Vaccinium corymbosum*)	Particulate matter-exposed rats	Blueberry anthocyanin-enriched extract	Administration of 0.5, 1, or 2 g/kg of extract, daily for 5 weeks	Improvement in abnormal ECG (higher with 1 g/kg) Reduction in cardiac injury biomarkers (higher with 1 g/kg)	[114]
Red raspberry (*Rubus idaeus*)	Dietary-induced obese rats	Freeze-dried fruit	High-fat and sucrose diet supplemented with 10% raspberry or 10% blackberry + raspberry	Reduction in inflammation and oxidative stress (alteration of redox proteins from the myocardium), when combined with blackberry	[110]
	Obese diabetic rats	Freeze-dried powder	Administration of 0.8 g of powder, daily for 8 weeks	Reduction in expression of proteins related to cardiac remodelling and oxidative and inflammatory stress No effects on heart lipid composition, adipokines, and morphology	[115]
	Spontaneously hypertensive rats	Ethyl acetate extract	Administration of 100 or 200 mg/kg of extract, daily for 5 weeks	Reduction in blood pressure (higher with high dose), MDA (with high dose), plasma endothelin (with high dose) Increase in nitric oxide (with low dose) and superoxide dismutase levels	[78]
	MetS rat models	Freeze-dried powder	Supplementation of the equivalent of 1 and ½ cups of fresh fruit in humans, daily for 8 weeks	Improvement in aorta vasoconstriction and vasorelaxation	[116]
Strawberry (*Fragaria × ananassa*)	n.f.				

AC = anthocyanins; n.f. = not found; MetS = metabolic syndrome; ECG = electrocardiogram; MDA = malondialdehyde.

**Table 4:** Clinical trials regarding the consumption of cherries and berries and its effects on cardiovascular disease-related factors.

Fruit (Species)	Subjects	Fruit Preparation	Procedure	Main results	Ref.
Tart cherry (*Prunus cerasus*)	Middle-aged adults (48 ± 6 yo)	Concentrate juice	Consumption of 30 mL concentrate in 240 mL water, 2× per day for 3 months	No effects on vascular function or metabolic health	[117]
	Older adults (65-80 yo)	Concentrate juice	Consumption of 68 mL concentrate in 412 mL water, daily for 12 weeks	Reduction in systolic blood pressure and LDL-C	[118,119]
	Healthy adults (18–65 yo)	Concentrate juice	Consumption of 30 mL concentrate in 300 mL water, 2× per day for 20 days	No effects on systolic blood pressure and anthropometric, energy expenditure, substrate oxidation, haematological, diastolic blood pressure/resting heart rate, psychological well-being, and sleep efficacy measurements	[120]
	MetS adults (49 ± 12 yo)	Capsules or concentrate juice	Consumption of 30 mL concentrate in 100 mL water or 10 capsules with 130 mL water, on different occasions	Reduction in systolic blood pressure	[66]
	Early hypertension men (31 ± 9 yo)	Concentrate juice	Consumption of 60 mL concentrate juice, once	Reduction in systolic blood pressure	[68]
	MetS adults (20–60 yo)	Juice	Consumption of 240 mL juice, 2× per day for 12 weeks	Reduction in cardiometabolic biomarkers	[121]
	Healthy adults (30–50 yo)	Concentrate juice	Consumption of 30 mL concentrate in 220 mL water, daily for 6 weeks	No effect on arterial stiffness, hsCRP, and cardiovascular disease markers Increase in plasma antioxidant capacity	[122]
	Healthy adults (18–65 yo)	Seed extract	Consumption of 250 mg extract, daily for 14 days	Reduction in circulating neutrophils and ferritin levels Increase in mean cell volume, serum transferrin, mean peroxidase index, and representation of peripheral blood lymphocytes	[123]
	MetS adults (50 ± 10 yo)	Concentrate juice	Consumption of 30 mL concentrate in 100 mL water	Reduction in systolic blood pressure and mean arterial pressure, total cholesterol, LDL-C, total-C:HDL-C ratio, and respiratory exchange ratio	[67]
Sweet cherry (*Prunus avium*)	Obese adults (≥18 yo)	Juice supplemented with powder	Consumption of 200 mL of juice, daily for 30 days	Reduction in systolic and diastolic blood pressure, and blood inflammatory biomarkers No effect on lipids	[71]
Blackberry (*Rubus* spp.)	Healthy adults	Juice	High-fat and high-carbohydrate diet supplemented with 250 mL of juice, 3× per day for 14 days	Reduction in plasma triglycerides, total cholesterol, and glucose levels No effect on LDL-C and HDL-C	[124]
Blueberry (*Vaccinium corymbosum*)	Healthy adults (18–60 yo)	Fresh fruit or freeze-dried powder	Consumption of 160 g of fresh fruit or 20 g of powder, daily for 1 week	No effect on blood pressure, endothelial function, plasma lipids, and nitrite levels Increase in plasma NO^2−^ levels	[125]
	MetS adults (50 ± 3 yo)	Freeze-dried powder	Consumption of 50 g powder in 480 mL water with vanilla extract or “Splenda”, daily for 8 weeks	Reduction in systolic diastolic blood pressure, oxidized LDL-C, and MDA No effects on lipid profiles	[72]
	MetS adults (50–75 yo)	Fresh fruit	Consumption of 75 or 150 g of blueberries, daily for 6 months	Improved endothelial function, systemic arterial stiffness, and reduced cyclic guanosine monophosphate concentrations (with high dose) No effects on pulse wave velocity, blood pressure, NO, and plasma thiol status	[126]
	MetS adults (>20 yo)	Freeze-dried powder	Consumption of 22.5 g of powder mixed into 29.6 mL yogurt and skim milk-based smoothie, 2× per day for 6 weeks	No effects on blood pressure Improvement in resting endothelial function	[127]
	MetS adults (63.4 ± 7.4)	Freeze-dried powder	Consumption of 26 g of powder with a 500 g energy-dense milkshake, once	Reduced cholesterol Increased HDL-C, fractions of HDL, and Apo-AI	[128]
	Sedentary adults (40–70 yo)	Freeze-dried powder	Consumption of 38 g of powder, daily for 7 days; consumption once, again, after 3 weeks	Reduced systolic blood pressure No effect on diastolic blood pressure	[73]
	Adults with MetS risk (22–53 yo)	Freeze-dried powder	Consumption of 25 g of powder in 300 mL water, 2× per day for 8 weeks	No effect on markers of cardiometabolic health Changed expression of 49 genes and abundance of 35 metabolites of immune-related pathways	[129]
	Pre- and stage 1-hypertensive postmenopausal women (45–65 yo)	Freeze-dried powder	Consumption of 11 g of powder in 240 mL, 2× per day for 8 weeks	Decreased one marker of oxidative DNA damage after 4 but not 8 weeks No effect on inflammation, and antioxidant defence biomarkers	[130]
	Hypertension postmenopausal women (45–65 yo)	Freeze-dried powder	Consumption of 11 g of powder in water, 2× per day for 12 weeks	Improved endothelial function No effects on blood pressure, arterial stiffness, blood biomarkers, and endothelial cell protein expression	[87]
	MetS adults (≥20 yo)	Freeze-dried powder	Consumption of 22.5 g of powder mixed into 356 mL yogurt and skim milk-based smoothie, 2× per day for 6 weeks	Reduction in oxidative stress and expression of inflammatory markers in monocytes	[88]
	Healthy women (30–50 yo)	Blueberry and raspberry pomace cookies	Consumption of 4 cookies (32 g), daily for 4 weeks	Reduction in LDL-C, ALT, and AST Increase in adiponectin levels	[96]
Red raspberry (*Rubus idaeus*)	Overweight pre-diabetic adults (20–60 yo)	Frozen fruit	Consumption of 125 or 250 g of fruit with a high-carbohydrate breakfast in 3 separate days	No effects on oxidative stress and inflammatory biomarkers	[131]
	Healthy women (30–50 yo)	Blueberry and raspberry pomace cookies	Consumption of 4 cookies (32 g), daily for 4 weeks	Reduction in LDL-C, ALT, and AST Increase in adiponectin levels	[96]
Strawberry (*Fragaria × ananassa*)	MetS adults (47 ± 3 yo)	Freeze-dried fruit beverage	Consumption of 50 g of powder with 4 cups of water, daily for 8 weeks	Reduction in total and LDL-C and circulating adhesion molecules	[132]
	Healthy adults (27 ± 3.2 yo)	Fresh fruit	Consumption of 500 g of fruit, daily for 1 month	Reduction in total cholesterol, LDL-C, and triglycerides levels and oxidative stress markers No effects on HDL-C	[95]
	Obese adults (20–50 yo)	Freeze-dried powder	Consumption of 80 g of powder mixed in a milkshake, yogurt, cream cheese, or water-based sweetened beverage, 2× per day for 3 weeks	Reduction in plasma cholesterol, small HDL particles, Increased LDL particle size	[133]
	Overweight or obese adults (28 ± 2 yo)	Freeze-dried powder	High-fat meal with 40 g of powder, once	No effects on vascular function and postprandial triglycerides	[134]
	Healthy male adolescents (14–18 yo)	Freeze-dried powder	Consumption of 25 g of powder with water, 2× per day for 1 week	Increase in reactive hyperaemia index	[135]
	Hyperlipidaemic adults (50.9 ± 15 yo)	Freeze-dried fruit beverage	Consumption of a 10 g freeze-dried fruit beverage, daily for 6 weeks + 3 moments of consumption of a high-fat diet	Reduction in postprandial triglycerides and oxidized LDL	[102]
	Obese adults (53 ± 13 yo)	Freeze-dried powder	Consumption of 32 g or 13 g of powder with water, daily for 4 weeks	Reduction in LDL, VLDL, and LDL particles, serum PAI-1 (with high dose) No effects on lipid profile	[136]
	Overweight adults (50.9 ± 15 yo)	Freeze-dried fruit beverage	Consumption of 10 g freeze-dried fruit beverage, daily for 6 weeks	Reduction in postprandial PAI-1, IL-1*β* No effects on platelet aggregation, hsCRP, TNF-*α*	[137]
	Moderate hypercholesteremia (53 ± 1 yo)	Freeze-dried fruit beverage	Consumption of 25 g freeze-dried fruit beverage, 2× per day for 4 weeks	Reduction in systolic blood pressure No effects on LDL, total cholesterol, triglycerides, hsCRP	[74]
	Healthy adults (20–60 yo)	Fruit pulp	Consumption of 500 g of pulp, daily for 30 days, followed by a washout period and new consumption period	Reduction in paraoxonase PON-1 activity No effects on the lipid profile	[138]
	Obese adults (49 ± 10 yo)	Freeze-dried powder	Consumption of 25 g or 50 g of powder in 474 mL of water, daily for 12 weeks	Increased plasma antioxidant biomarkers (gluthatione higher with high dose vs low and serum catalase higher with low dose)	[139]
	Overweight or obese adults (50 ± 1.0 yo)	Freeze-dried powder	Consumption of 13 g or 40 g of powder in water, daily for 4 weeks	Reduction in total cholesterol (with low dose) No effects on vascular function, inflammation, or HDL efflux	[140]
	Overweight or obese adult males (31.5 yo)	Freeze-dried powder	Consumption of 25 g of powder in a high-carbohydrate meal, 16 h after 40 min of intense physical exercise	Reduction in postprandial lipaemia No effects on postprandial triglycerides and lipid-related oxidative stress markers	[141]
	Diabetic women (51.57 ± 10 yo)	Freeze-dried powder	Consumption of 25 g of powder in water, 2× per day for 6 weeks	Improvement in glycaemic control and antioxidant status Reduction in lipid peroxidation and inflammatory response No effects on serum glucose and anthropometric indices	[142]
	Adults with abdominal adiposity and high serum lipids (49 ± 10 yo)	Freeze-dried powder	Consumption of 25 g or 50 g of powder in 474 mL of water, daily for 12 weeks	Reduction in total and LDL-C, LDL particles, lipid peroxidation (higher with high dose) No effects on adiposity, blood pressure, glycaemic control, or inflammation	[143]
	Pre- and stage 1-hypertensive postmenopausal women (45–65 yo)	Freeze-dried powder	Consumption of 12.5 or 25 g of powder in 240 mL, 2× per day for 8 weeks	No effects on blood pressure or vascular function	[60]

yo = years old; MetS = metabolic syndrome; LDL = low-density lipoprotein; LDL-C = low-density lipoprotein cholesterol; HDL = high-density lipoprotein; HDL-C = high-density lipoprotein cholesterol; VLDL = very low-density lipoprotein; total-C = total cholesterol; MDA = malondialdehyde; ALT = alanine aminotransferase; AST = aspartate aminotransferase; PAI-1 = plasminogen activator inhibitor-1; hsCRP = high-sensitivity C-reactive protein; TNF-*α* = tumour necrosis factor; PON-1 = paraoxonase 1; IL-1*β* = interleukin 1 beta; Apo-AI = Apolipoprotein A1; NO = nitric oxide.

**Figure 3:**
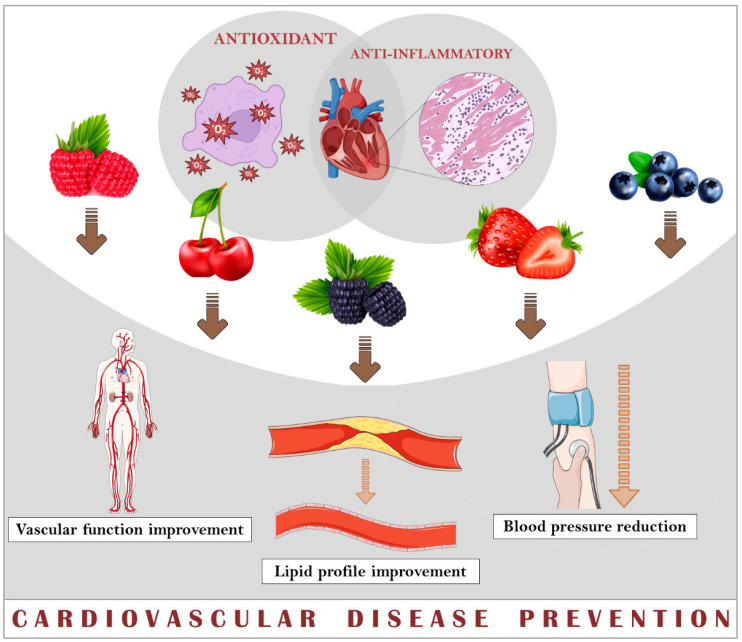
Preventive cardiovascular disease effects of cherries and berries.

Research suggests several mechanisms for the prevention and/or treatment of CVD, but these mechanisms are hard to be precise, due to the pleiotropic effect of these compounds [89]. Despite the potential of cherries and berries to reduce risk factors related to CVD, such as blood pressure, endothelial function, and lipid profiles (Figure 3), some of these studies have not yet shown a beneficial effect of fruit consumption on these parameters, and the results are not sufficient to confirm a therapeutic potential. Further research is needed to investigate the optimal dose, duration, and form of fruit consumption to maximise the health benefits. 

One of the most important aspects that has a major impact on the outcomes of this research is the type of preparation, as it affects the compounds available in the fruit. Juices and concentrates are popular fruit preparations due to their convenience and palatability [144]. However, the processing involved in juice extraction and concentration can result in the loss of certain nutrients and phytochemicals, especially fibre [145]. In addition, concentrates often contain higher concentrations of sugars and calories, which may counteract some of the health benefits associated with fruit consumption. Whole fruits should be chosen whenever possible to ensure adequate fibre intake [146].

Freeze-dried fruit powders offer a convenient and concentrated source of nutrients and phytochemicals. The freeze-drying process preserves the fruit’s flavour, colour, and nutritional content, making it an attractive option for supplementation or incorporation into various food products [147]. In addition, this type of preparation can limit the seasonal variation issues associated with fresh fruit, as the properties of freeze-dried fruit are preserved for a longer period and the same composition can be used throughout the year. However, it is important to note that some heat-sensitive nutrients and antioxidants can still be lost during processing. 

Ground fruit seeds are often used as supplements or added to food preparations. Incorporating seed powders in the diet can provide additional nutrients and phytochemicals, and they have been identified as an important source of antioxidants, demonstrating higher antioxidant activity than the edible parts of the fruit [148,149]. However, it is important to note that excessive consumption of seed powder may not be suitable for everyone, particularly those with certain dietary restrictions or allergies [150].

Fruit pulp, whether consumed in its natural form or as an ingredient in various food preparations offers the benefits of the whole fruit, including fibre, vitamins, minerals, and phytochemicals. However, the processing and cooking methods can affect the stability and bioavailability of certain nutrients and phytochemicals [151].

Consumption frequency and quantity also seem to be an important factor. Regular and constant intake over a period of weeks or months appears to be better than one single or irregular intake. For instance, compared to one-time or irregular doses, regular daily use of blueberry powder over several weeks has greater outcomes in terms of antioxidant effects and blood pressure lowering (Table 4). Some studies compare the effect of different doses of the same fruit. In rat models, 10% blackberry supplementation led to the downregulation of inflammation genes and reduction of weight gain, while a 5% dose did not [112]; administration of 200 mg/kg of red raspberry extract rather than 100 mg/kg improved blood pressure, and plasma endothelin levels (however, only the 100 mg/kg dose increased nitric oxide levels) [78]; regarding blueberries, the concentration of 1 g/kg was more effective in improving ECG and reducing cardiac injury biomarkers than 0.5 and 2 g/kg [114] (Table 3). Clinical trials revealed that only a high dose of blueberry (150 g vs. 75 g) improved endothelial function, arterial stiffness, and guanosine monophosphate concentrations in metabolic syndrome adults [126]; strawberry powder’s higher doses showed better results in lipid profiles in two studies [136,143], while another study showed benefits only with the lowest dose tested [140] (Table 4). While there seems to be a tendency for greater effects with higher amounts of fruit, the heterogeneous results do not allow us to confirm this relationship. 

When comparing the populations, elderly and metabolic syndrome humans seem to show more pronounced benefits, often manifesting improvements in blood pressure and metabolic biomarkers. Healthy adults frequently show no significant changes in parameters like blood pressure and lipid profiles. This indicates the potential of this kind of treatment to help subjects with a compromised health status.

However, it is also important to assess the safe doses for consumption of these bioactive compounds. Anthocyanins do not appear to be toxic when consumed in a normal amount; an LD_50_ of 25,000 mg in mice and 20,000 mg in rats per kg of body weight (bw) has been defined with no adverse effects, while, in rabbits, the blood pressure was not affected after oral administration of 6g/kg bw of anthocyanin glycosides; in guinea pigs and dogs it has been reported that a dose of 3 g/kg bw does not cause subchronic toxicity [152]. Many studies mention the use of approximately one or two cups of berries (or a dose of a formulation that is equivalent to this amount) [126,128,131]. Basu et al. claimed that a dose of 50 g of blueberries, which is equivalent to approximately 350 g or 2.3 cups, was well tolerated by the metabolic syndrome subjects in their trial [72]. This dose is in accordance with the WHO recommendation of a consumption of 400 g of fruits and vegetables per day [153]. However, further studies on a variety of compounds and doses in human cells are needed to ensure the safety of this consumption.

## 5. Limitations and Future Perspectives

The limitations of this type of study are related to the difficulty of assessing the relationship between fruit consumption and health outcomes. The first challenge is the natural variability of fruits. Fruit composition can be strongly influenced by a variety of factors, including location, climate, and agricultural practices. This can affect the nutritional value and potential health benefits of the fruit. Fruit preparation (concentrates, juices, freeze-dried) is another important factor, as fruits can be subjected to processing that changes their composition, potentially affecting their bioactive compounds and nutrient content.

The small sample sizes of some studies may affect the statistical significance. In addition, short intervention periods may not capture the long-term effects of fruit consumption on health outcomes. Such limitations emphasize the need for larger, longer-term studies to provide stronger evidence. Furthermore, not all studies can adequately control for factors such as diet, physical activity, and medication use, which can interfere with the associations between fruit consumption and health outcomes, hiding the true effect of the fruit. 

Another challenge is ensuring that participants adhere to fruit consumption interventions, which requires strategies to monitor and encourage compliance throughout the duration of the study. Finally, studies that focus on specific populations or geographical regions further limit the generalizability of the results. Findings from homogeneous populations may not apply to more diverse or representative populations, highlighting the need for studies conducted across varied demographic and geographic contexts. Therefore, long-term prospective studies are essential in the future to better understand the cumulative effects of fruit consumption on chronic disease risk and overall health outcomes. Studies with larger sample sizes and diverse study populations should be prioritized. 

It is important to evaluate the underlying mechanisms by which specific fruits or their bioactive compounds exert beneficial effects, using experimental models and biomarker analyses. The synergistic effects of fruits in the context of overall dietary patterns (e.g., Mediterranean diet) are also important. Finally, the results of this type of research need to be translated into dietary recommendations and public health policies to promote fruit consumption as part of a healthy diet.

## 6. Conclusions

The consumption of cherries and berries has significant potential to reduce the risk factors associated with CVD, which remains a leading cause of mortality worldwide. The rich selection of bioactive compounds present in these fruits, including phenolic acids, flavonoids, and anthocyanins, offer multiple health benefits ranging from blood pressure reduction to lipid profile improvements as well as the enhancement of endothelial function, due to their capacity to attenuate inflammation and oxidative stress. However, despite the promising findings from various in vivo studies and clinical trials, several challenges and limitations can be found when assessing the relationship between fruit consumption and health outcomes, namely, the variability of the fruits themselves, influenced by factors such as location, climate, and agricultural practices, which can affect their nutritional value and bioactive compound content, to the type of fruit preparation, whether juice, concentrate, or freeze-dried, which can alter the composition of bioactive compounds, and potentially affect their health benefits. In addition, limitations in study design, such as small sample sizes, short duration of intervention, and inadequate control of confounding factors, can affect the strength of the evidence. To address these challenges, longer-term studies with diverse study populations are needed to better understand the cumulative effects of fruit consumption on chronic disease risk and overall health outcomes. These studies should prioritize the evaluation of the underlying mechanisms by which specific fruits or their bioactive compounds exert beneficial effects, using experimental models and biomarker analyses. By overcoming these barriers, it is possible to improve our understanding of the role of cherries and berries in cardiovascular health, not only benefiting individuals by enhancing their well-being but also improving strategies for the prevention and management of CVD on a global scale.

## Figures and Tables

**Table 1:** Nutritional composition of cherries and berries (cherry, blackberry, blueberry, strawberry, raspberry) [8].

	Cherry	Blackberry	Blueberry	Strawberry	Raspberry
	Amount per 100 g of edible portion				
Energy (kcal)	67.00	43.00	43.00	34.00	49.00
Lipids (g)	0.70	0.90	0.60	0.40	0.60
Saturated fatty acids (g)	0.20	0.00	0.10	0.00	0.00
MUFA (g)	0.20	0.00	0.10	0.10	0.10
PUFA (g)	0.20	0.30	0.20	0.20	0.30
Linoleic acid (g)	0.20	0.20	0.12	0.10	0.20
Trans fatty acids (g)	0.00	0.00	0.00	0.00	0.00
Carbohydrates (g)	13.30	4.50	6.40	5.30	5.10
Sugar (g)	13.30	4.20	6.40	5.30	5.10
Oligosaccharides (g)	0.00	0.00	0.00	0.00	0.00
Fibre (g)	1.60	4.60	3.10	2.00	6.70
Protein (g)	0.80	1.40	0.50	0.60	0.90
Salt (g)	0.00	0.00	0.00	0.00	0.00
Water (g)	82.60	88.00	87.00	90.10	84.30
Organic acids (g)	0.40	0.90	1.40	0.80	1.90
Cholesterol (mg)	0.00	0.00	0.00	0.00	0.00
Vitamin A (µg)	24.00	27.00	8.00	4.00	2.00
Carotene (µg)	141.00	164.00	47.00	26.00	10.00
Vitamin D (µg)	0.00	0.00	0.00	0.00	0.00
α-tocopherol (mg)	0.13	4.42	1.90	0.20	0.20
Thiamine (mg)	0.04	0.02	0.04	0.03	0.03
Riboflavin (mg)	0.06	0.04	0.07	0.06	0.02
Niacin (mg)	0.20	0.54	0.42	0.60	0.60
Tryptophan/60 (mg)	0.10	0.20	0.05	0.20	0.20
Vitamin B6 (mg)	0.04	0.05	0.06	0.05	0.05
Vitamin B12 (µg)	0.00	0.00	0.00	0.00	0.00
Vitamin C (mg)	6.00	16.50	0.15	0.47	0.30
Pholate (µg)	5.00	25.00	11.5	47.00	0.33
Ash (g)	0.43	0.40	0.25	0.58	0.54
Sodium (mg)	1.00	1.80	0.30	2.00	1.00
Potassium (mg)	210.00	240.00	110.00	140.00	230.00
Calcium (mg)	14.00	28.00	19.00	25.00	26.00
Phosphorous (mg)	15.00	33.00	20.00	26.00	23.00
Magnesium (mg)	10.00	22.00	9.00	10.00	20.00
Iron (mg)	0.40	0.60	0.60	0.80	0.50
Zinc (mg)	0.10	0.50	0.20	0.10	0.30
Selenium (µg)	n.a.	0.10	0.10	n.a.	n.a.
Iodol (µg)	n.a.	0.40	1.00	3.80	n.a.

n.a. = not available.
